# Trends in invasive bacterial diseases during the first 2 years of the COVID-19 pandemic: analyses of prospective surveillance data from 30 countries and territories in the IRIS Consortium

**DOI:** 10.1016/S2589-7500(23)00108-5

**Published:** 2023-07-27

**Authors:** David Shaw, Raquel Abad, Zahin Amin-Chowdhury, Adriana Bautista, Desiree Bennett, Karen Broughton, Bin Cao, Carlo Casanova, Eun Hwa Choi, Yiu-Wai Chu, Heike Claus, Juliana Coelho, Mary Corcoran, Simon Cottrell, Robert Cunney, Lize Cuypers, Tine Dalby, Heather Davies, Linda de Gouveia, Ala-Eddine Deghmane, Walter Demczuk, Stefanie Desmet, Mirian Domenech, Richard Drew, Mignon du Plessis, Carolina Duarte, Helga Erlendsdóttir, Norman K Fry, Kurt Fuursted, Thomas Hale, Desiree Henares, Birgitta Henriques-Normark, Markus Hilty, Steen Hoffmann, Hilary Humphreys, Margaret Ip, Susanne Jacobsson, Christopher Johnson, Jillian Johnston, Keith A Jolley, Aníbal Kawabata, Jana Kozakova, Karl G Kristinsson, Pavla Krizova, Alicja Kuch, Shamez Ladhani, Thiên-Trí Lâm, María Eugenia León, Laura Lindholm, David Litt, Martin C J Maiden, Irene Martin, Delphine Martiny, Wesley Mattheus, Noel D McCarthy, Mary Meehan, Susan Meiring, Paula Mölling, Eva Morfeldt, Julie Morgan, Robert Mulhall, Carmen Muñoz-Almagro, David Murdoch, Joy Murphy, Martin Musilek, Alexandre Mzabi, Ludmila Novakova, Shahin Oftadeh, Amaresh Perez-Argüello, Maria Pérez-Vázquez, Monique Perrin, Malorie Perry, Benoit Prevost, Maria Roberts, Assaf Rokney, Merav Ron, Olga Marina Sanabria, Kevin J Scott, Carmen Sheppard, Lotta Siira, Vitali Sintchenko, Anna Skoczyńska, Monica Sloan, Hans-Christian Slotved, Andrew J Smith, Anneke Steens, Muhamed-Kheir Taha, Maija Toropainen, Georgina Tzanakaki, Anni Vainio, Mark P G van der Linden, Nina M van Sorge, Emmanuelle Varon, Sandra Vohrnova, Anne von Gottberg, Jose Yuste, Rosemeire Zanella, Fei Zhou, Angela B Brueggemann

**Affiliations:** a Nuffield Department of Population Health, Big Data Institute, University of Oxford, Oxford, UK; b National Reference Laboratory for Meningococci, National Center of Microbiology, Instituto de Salud Carlos III, Madrid, Spain; c Immunisation and Countermeasures Division, UK Health Security Agency, London, UK; d Instituto Nacional de Salud, Bogotá, Colombia; e Irish Meningitis and Sepsis Reference Laboratory, Children's Health Ireland, Dublin, Ireland; f Staphylococcus and Streptococcus Reference Section, AMRHAI, UK Health Security Agency, London, UK; g Department of Pulmonary and Critical Care Medicine, Center of Respiratory Medicine, National Clinical Research Center for Respiratory Diseases, Institute of Respiratory Medicine, Chinese Academy of Medical Sciences, Peking Union Medical College, Beijing, China; h Swiss National Reference Center for Invasive Pneumococci, Institute for Infectious Diseases, University of Bern, Bern, Switzerland; i Department of Pediatrics, Seoul National University College of Medicine, Seoul, South Korea; j Department of Health, Microbiology Division, Public Health Laboratory Services Branch, Centre for Health Protection, Hong Kong Special Administrative Region, China; k University of Würzburg, Institute for Hygiene and Microbiology, National Reference Centre for Meningococci and Haemophilus influenzae, Würzburg, Germany; l Department of Microbiology, Royal College of Surgeons in Ireland, Dublin, Ireland; m Public Health Wales, Cardiff, Wales, UK; n National Reference Centre for Streptococcus pneumoniae, University Hospitals Leuven, Leuven, Belgium; o Department of Microbiology, Immunology and Transplantation, KU Leuven, Leuven, Belgium; p Statens Serum Institut, Department of Infectious Disease Epidemiology & Prevention, Copenhagen, Denmark; q Meningococcal Reference Laboratory, Institute of Environmental Science and Research, Porirua, New Zealand; r Centre for Respiratory Diseases and Meningitis, National Institute for Communicable Diseases, Division of the National Health Laboratory Service, Johannesburg, South Africa; s Institut Pasteur, Univeristé Paris Cité, Invasive Bacterial Infections Unit and National Reference Centre for Meningococci and Haemophilus influenzae, Paris, France; t National Microbiology Laboratory, Public Health Agency of Canada, Winnipeg, MB, Canada; u National Center for Microbiology and CIBER of Respiratory Research, Instituto de Salud Carlos III, Madrid, Spain; v Clinical Innovation Unit, Rotunda, Dublin, Ireland; w Department of Clinical Microbiology, Landspitali, The National University Hospital of Iceland, Reykjavik, Iceland; x Immunisation and Vaccine Preventable Diseases Division and Respiratory and Vaccine Preventable Bacteria Reference Unit, UK Health Security Agency, London, UK; y Statens Serum Institut, Department of Bacteria, Parasites & Fungi, Copenhagen, Denmark; z Blavatnik School of Government, University of Oxford, Oxford, UK; aa Microbiology Department, Institut Recerca Sant Joan de Déu, Hospital Sant Joan de Deu, Barcelona, Spain; ab CIBER of Epidemiology and Public Health, Madrid, Spain; ac Karolinska Institutet, Karolinska University Hospital, Public Health Agency of Sweden, Stockholm, Sweden; ad Department of Clinical Microbiology, Beaumont Hospital, Dublin, Ireland; ae Department of Microbiology, Faculty of Medicine, The Chinese University of Hong Kong, Hong Kong Special Administrative Region, China; af National Reference Laboratory for Neisseria meningitidis, Department of Laboratory Medicine, Clinical Microbiology, Faculty of Medicine and Health, Örebro University, Örebro, Sweden; ag Public Health Agency, Belfast, UK; ah Department of Biology, University of Oxford, Oxford, UK; ai Laboratorio Central de Salud Pública, Asunción, Paraguay; aj National Reference Laboratory for Streptococcal Infections, Centre for Epidemiology and Microbiology, National Institute of Public Health, Prague, Czech Republic; ak National Reference Laboratory for Meningococcal Infections, Centre for Epidemiology and Microbiology, National Institute of Public Health, Prague, Czech Republic; al National Reference Centre for Bacterial Meningitis, Department of Epidemiology and Clinical Microbiology, National Medicines Institute, Warsaw, Poland; am Finnish Institute for Health and Welfare, Helsinki, Finland; an Respiratory and Vaccine Preventable Bacteria Reference Unit, UK Health Security Agency, London, UK; ao National Belgian Reference Centre for Haemophilus influenzae, Laboratoire des Hôpitaux Universitaires de Bruxelles-Universitair Laboratorium van Brussel, Brussels, Belgium; ap Faculty of Medicine and Pharmacy, University of Mons, Mons, Belgium; aq Meningococcal National Reference Centre, Sciensano, Belgium; ar Population Health Medicine, Public Health and Primary Care, Trinity College Dublin, Dublin, Ireland; as Division of Public Health Surveillance and Response, National Institute for Communicable Diseases, Division of the National Health Laboratory Service, Johannesburg, South Africa; at Public Health Agency of Sweden, Solna, Sweden; au Streptococcal Reference Laboratory, Institute of Environmental Science and Research Limited, Porirua, New Zealand; av Medicine Department, Universitat Internacional de Catalunya, Barcelona, Spain; aw University of Otago, Christchurch, New Zealand; ax Laboratoire National de Sante, Dudelange, Luxembourg; ay Ministère de la Santé - Direction de la santé, Luxembourg, Luxembourg; az National Reference Laboratory for Haemophilus Infections, Centre for Epidemiology and Microbiology, National Institute of Public Health, Prague, Czech Republic; aaa NSW Pneumococcal Reference Laboratory, Institute of Clinical Pathology and Medical Research - NSW Health Pathology, Sydney, NSW, Australia; aab Laboratorio de Referencia e Investigación en Resistencia a Antibióticos e Infecciones Relacionadas con la Asistencia Sanitaria, Centro Nacional de Microbiología, Instituto de Salud Carlos III, Majadahonda, Madrid, Spain; aac CIBER de Enfermedades Infecciosas, Instituto de Salud Carlos III, Madrid, Spain; aad National Belgian Reference Centre for Haemophilus influenzae, Laboratoire des Hôpitaux Universitaires de Bruxelles-Universitair Laboratorium van Brussel, Brussels, Belgium; aae Public Health Laboratories–Jerusalem, Public Health Services, Ministry of Health, Jerusalem, Israel; aaf Bacterial Respiratory Infection Service, Scottish Microbiology Reference Laboratories, Glasgow Royal Infirmary, Glasgow, UK; aag NSW Pneumococcal Reference Laboratory, Institute of Clinical Pathology and Medical Research - NSW Health Pathology, Sydney, NSW, Australia; aah Sydney Institute for Infectious Diseases, University of Sydney, NSW, Australia; aai Bacterial Respiratory Infection Service, Scottish Microbiology Reference Laboratories, Glasgow Royal Infirmary, Glasgow, UK; aaj College of Medical, Veterinary & Life Sciences, University of Glasgow, Glasgow, UK; aak Centre for Infectious Disease Control, National Institute for Public Health and the Environment, Bilthoven, Netherlands; aal National Meningitis Reference Laboratory, Department of Public Health Policy, School of Public Health, University of West Attica, Athens, Greece; aam Department of Medical Microbiology, German National Reference Centre for Streptococci, University Hospital RWTH Aachen, Aachen, Germany; aan Department of Medical Microbiology and Infection Prevention, and Netherlands Reference Laboratory for Bacterial Meningitis, Amsterdam University Medical Center, University of Amsterdam, Amsterdam, Netherlands; aao Laboratory of Medical Biology and National Reference Centre for Pneumococci, Intercommunal Hospital of Créteil, Créteil, France; aap National Laboratory for Meningitis and Pneumococcal Infections, Center of Bacteriology, Institute Adolfo Lutz, São Paulo, Brazil

## Abstract

**Background:**

The Invasive Respiratory Infection Surveillance (IRIS) Consortium was established to assess the impact of the COVID-19 pandemic on invasive diseases caused by *Streptococcus pneumoniae, Haemophilus influenzae, Neisseria meningitidis*, and *Streptococcus agalactiae*. We aimed to analyse the incidence and distribution of these diseases during the first 2 years of the COVID-19 pandemic compared to the 2 years preceding the pandemic.

**Methods:**

For this prospective analysis, laboratories in 30 countries and territories representing five continents submitted surveillance data from Jan 1, 2018, to Jan 2, 2022, to private projects within databases in PubMLST. The impact of COVID-19 containment measures on the overall number of cases was analysed, and changes in disease distributions by patient age and serotype or group were examined. Interrupted time-series analyses were done to quantify the impact of pandemic response measures and their relaxation on disease rates, and autoregressive integrated moving average models were used to estimate effect sizes and forecast counterfactual trends by hemisphere.

**Findings:**

Overall, 116 841 cases were analysed: 76 481 in 2018–19, before the pandemic, and 40 360 in 2020–21, during the pandemic. During the pandemic there was a significant reduction in the risk of disease caused by *S pneumoniae* (risk ratio 0·47; 95% CI 0·40–0·55), *H influenzae* (0·51; 0·40–0·66) and *N meningitidis* (0·26; 0·21–0·31), while no significant changes were observed for *S agalactiae* (1·02; 0·75–1·40), which is not transmitted via the respiratory route. No major changes in the distribution of cases were observed when stratified by patient age or serotype or group. An estimated 36 289 (95% prediction interval 17 145–55 434) cases of invasive bacterial disease were averted during the first 2 years of the pandemic among IRIS-participating countries and territories.

**Interpretation:**

COVID-19 containment measures were associated with a sustained decrease in the incidence of invasive disease caused by *S pneumoniae, H influenzae*, and *N meningitidis* during the first 2 years of the pandemic, but cases began to increase in some countries towards the end of 2021 as pandemic restrictions were lifted. These IRIS data provide a better understanding of microbial transmission, will inform vaccine development and implementation, and can contribute to health-care service planning and provision of policies.

**Funding:**

Wellcome Trust, NIHR Oxford Biomedical Research Centre, Spanish Ministry of Science and Innovation, Korea Disease Control and Prevention Agency, Torsten Söderberg Foundation, Stockholm County Council, Swedish Research Council, German Federal Ministry of Health, Robert Koch Institute, Pfizer, Merck, and the Greek National Public Health Organization.

**Research in context**

**Evidence before this study**

We searched PubMed, bioRxiv, and medRxiv for articles written in English and published before Dec 31, 2019, that reported on large-scale containment measures implemented during a pandemic. Search terms included “pandemic” AND “microbial transmission” OR “transmission” AND “containment”. Overall, 262 papers were identified, but none met our inclusion criteria. In the early stages of the COVID-19 pandemic (ie, January–May, 2020), the Invasive Respiratory Infection Surveillance (IRIS) Consortium reported a significant reduction in invasive disease due to bacterial pathogens transmitted via the respiratory route. In particular, infections due to *Streptococcus pneumoniae* decreased by 68% at 4 weeks after COVID-19 containment measures were imposed (incidence rate ratio 0·32 [95% CI 0·27–0·37]), and by 82% at 8 weeks (0·18 [0·14–0·23]). This reduction in disease was found to be associated with the implementation of COVID-19 stringency measures and changes in human social behaviour. All 26 countries and territories participating in IRIS reported a substantial reduction in infections during this period compared with the previous 2 years.

**Added value of this study**

These new data from the expanded IRIS Consortium (which comprises 30 countries and territories as of 2021) showed a sustained reduction in invasive disease throughout the first 2 years of the COVID-19 pandemic. Using time-series modelling, we estimated that more than 36 000 cases of invasive bacterial disease were averted in 2020–21 among the countries participating in IRIS; however, minor increases in disease cases in the latter half of 2021 require close monitoring to understand the nature of and possible reasons for re-emerging cases.

**Implications of all the available evidence**

Future epidemics and pandemics will occur and, although it is essential to understand the pathogen that is directly responsible for the pandemic, we need to understand that overall human health and the transmission of other microbes are also broadly affected by population-level responses to an epidemic or pandemic. Data from IRIS provide evidence of the effects of such public health responses on severe invasive bacterial infections across many countries during the COVID-19 pandemic.

## Introduction

Three of the most common causes of invasive bacterial disease are *Streptococcus pneumoniae, Haemophilus influenzae*, and *Neisseria meningitidis*; young children, adolescents, and older adults are at greatest risk of disease. All three bacterial species colonise the oropharynx or nasopharynx of healthy individuals and all three bacteria are transmitted person to person via respiratory droplets.

The Global Burden of Diseases, Injuries, and Risk Factors Study estimated that, in 2019, *S pneumoniae* was the leading bacterial cause of death among children younger than 5 years worldwide (225 000 deaths; 95% uncertainty interval [UI] 180 000–281 000).^1^ It was also the leading cause of deaths due to lower respiratory infections (653 000; 95% UI 553 000–777 000) and meningitis (44 500; 34 700–59 800) among people of all ages, and led to 40·3 million (32·8–50·0) years of life lost globally.^1^ The same report estimated that 101 000 deaths (95% UI 82 800–124 000) worldwide were due to *H influenzae*, the majority of which were due to lower respiratory infections, and an estimated 141 000 deaths (96 800–203 000) were from bloodstream infections and meningitis caused by *N meningitidis. N meningitidis* is a global pathogen but a particular public health problem in Africa since it is a cause of meningitis epidemics, both within and outside the meningitis belt.^2^ *S pneumoniae* and *H influenzae* are also among the most common causes of deaths associated with infections caused by bacteria that are resistant to antibiotics.^3^

The Invasive Respiratory Infection Surveillance (IRIS) Consortium, an international network of microbiology laboratories in 30 countries and territories, was established early in 2020 in response to the COVID-19 pandemic and concerns about the potential for increased post-viral secondary bacterial infections.^4^ The main aim of the IRIS Consortium is to investigate the incidence of invasive diseases caused by *S pneumoniae, H influenzae*, and *N meningitidis*. Invasive infections due to these bacterial species are legally notifiable to public health registries in the majority of countries participating in IRIS.^4^

Previously, we reported significant reductions in the incidence of diseases caused by all three bacteria early in the COVID-19 pandemic and showed that these reductions were associated with the timing and stringency of COVID-19 containment measures.^4^, ^5^, ^6^, ^7^, ^8^, ^9^ A subset of laboratories also submitted data for cases of disease caused by *Streptococcus agalactiae*, a major cause of invasive disease across all age groups but especially neonates, and which is not transmitted via the respiratory route.^1^, ^10^ *S agalactiae* was included as a comparator organism to assess the stability of routine disease surveillance during the pandemic. There was no change in the incidence of invasive *S agalactiae* infections in the early months of the pandemic, suggesting that any disruptions to routine laboratory surveillance during the COVID-19 pandemic were minor and did not explain the observed reductions in diseases caused by *S pneumoniae, H influenzae*, and *N meningitidis*.^4^

We conducted an expanded prospective analysis of surveillance data for four bacterial species (*S pneumoniae, H influenzae, N meningitidis*, and *S agalactiae*) in the 2 years before COVID-19 (2018–19) and the first 2 years of the pandemic (2020–21). Four countries were added to the IRIS Consortium since our first publication in 2021,^4^ which expanded the geographical coverage of IRIS to 30 countries and territories across six continents. We collected data on patient age and bacterial serotype or group to assess epidemiological changes that might have implications for disease burden and vaccination programmes. We quantified the effect of COVID-19 restrictions on the four pathogens under investigation, utilised time-series modelling techniques to analyse changes in disease during the first 2 years of the pandemic, and estimated the number of cases averted. We also assessed the incidence of disease by patient age and serotype or group, to investigate whether the epidemiology of invasive disease had changed during 2020–21 compared to the pre-pandemic years.

## Methods

### Study design and participants

For this prospective analysis, national reference and expert microbiology laboratories in 30 countries and territories submitted data on confirmed cases of invasive infections (within a normally sterile site) caused by one or more of the four bacteria under investigation. Australia, Colombia, Greece, and Paraguay joined IRIS after the initial establishment of the consortium. All IRIS-participating laboratories provided national reference data apart from Australia (New South Wales only) and China (one Beijing hospital only). Data were collected for patients of all ages except in South Korea, where only data from patients aged younger than 16 years were available for analyses.

### Data collection

No patient-identifiable data were submitted to IRIS. In the originating laboratories, bacteria from clinical samples were primarily recovered and identified by standard microbiological culture methods and occasionally by PCR testing. Invasive disease cases identified from Jan 1, 2018, to Jan 2, 2022 (the end of ISO [International Organization for Standardization] week 52 for 2021), were included in the current analyses. The PubMLST suite of databases was used to collect and manage IRIS data, and a private project only accessible to IRIS participants was used for each of the four organisms. At a minimum, information about the specimen sampling date, patient age, and serotype or group was submitted for each case except where data protection rules in a country prevented the submission of data on patient age. Study data were entered by IRIS participants or the database curators (ABB, KAJ, and DS). Automated data integrity checks were applied before data upload, and all IRIS data were manually checked by the curators for data consistency; any discrepant or missing data were queried and resolved with the submitting laboratory.

Google COVID-19 Community Mobility Reports (CCMRs) are anonymised, within-country mobile device location history data that capture the movement of people in six categories, including time spent in workplaces and residential areas. Google CCMR data are calculated as a daily percentage change from the baseline day, which was the median value between Jan 3 and Feb 6, 2020. In our previous study we used Google CCMR data to estimate the week when each country first implemented COVID-19 containment measures. We used the same estimates in the current analyses, and for the four additional countries the week of implementation was calculated as described previously.^4^

The stringency of each country's COVID-19 containment measures was quantified with the Oxford Blavatnik COVID-19 Government Response Tracker (OxCGRT).^11^ This stringency index combines nine indicators that are tracked daily: school, workplace, and public transport closures; public event cancellations; gathering restrictions; stay at home requirements; internal movement restrictions; international travel controls; and public information campaigns. A composite stringency index variable between 0 and 100 is calculated and is available for download on the OxCGRT website. For our analyses, the daily stringency index was converted into an ISO weekly index by taking the mean stringency index metric for that week. Cumulative weekly case counts for each organism were plotted against the weekly stringency index for each country and organism.

### Time-series analysis and decomposition

Case counts were summed by month to generate country-specific and organism-specific time series for 2018–21. Monthly case totals were used to improve the overall model fit and accommodate the 2020 leap year (ie, 53 weeks). A second time-series analysis was done for the *S pneumoniae, H influenzae*, and *N meningitidis* datasets to account for potential seasonal differences, whereby case counts were pooled by countries residing in the northern or southern hemispheres, respectively. *S agalactiae* data were only collected from countries in the northern hemisphere.

### Interrupted time-series analysis

Seasonal autoregressive integrated moving average (ARIMA) models were used to quantify the impact of COVID-19 containment measures on the incidence of invasive bacterial disease, and to generate counterfactual trends with 95% prediction intervals (PIs) for each of the four pathogens. Although it would have been desirable to fit separate ARIMA models by country, the dataset in most countries was too small for this to be implemented, and residual autocorrelation and negative case counts produced after model fitting precluded this approach.

ARIMA models took the simplified form of:

$$ARIMA\left(p,d,q\right){\left(P,D,Q\right)}_{s}$$

where *p*=non-seasonal autoregressive (AR) order, *d*=non-seasonal differencing, *q*=non-seasonal moving average (MA) order, *P*=seasonal AR order, *D*=seasonal differencing, *Q*=seasonal MA order, and *s*=time span of repeating seasonal pattern (number of observations in a year), 12 months.

Box-Jenkins methodology was applied when building the ARIMA model and both manual and automated methods were used to select the final models.^12^ Each time series was assessed for stationarity with the augmented Dickey-Fuller unit root test, which tested the null hypothesis of non-stationarity.^12^ None of the individual time series analysed in this study was non-stationary so no adjustments were required.

Manual model identification and coefficient estimation were performed, and we accounted for any seasonal pattern in the time series. We utilised the autocorrelation function and the partial autocorrelation function to decide on the potential inclusion of and number of autoregressive or moving average components (order selection), or both. The final ARIMA models were selected with a combination of unit root tests, maximum likelihood estimation, and minimised corrected Akaike information criterion values.^12^, ^13^

To measure the impact of COVID-19 containment measures, step and slope variables were included as regressors in the final ARIMA models: step (0 before containment measure implementation, 1 thereafter); and slope (0 before containment measure implementation, +1 for each month thereafter). Based on Google CCMR data, the step variable switched from 0 to 1 from March, 2020, onwards for all countries.^4^ These changes reflect the change in personal behaviour in response to the pandemic as well as the containment measures initiated across the 30 countries and territories in our cohort. The final ARIMA models were used to produce a counterfactual prediction that assumed the COVID-19 pandemic did not occur, based on disease data from the two pre-pandemic years, which generated a mean monthly case estimate and 95% PI. The relative risk (RR) of invasive disease, and number of cases averted, from March, 2020 (when the pandemic was officially declared by WHO), were estimated as follows: RR=number of cases observed / number of counterfactual cases. The major threats to internal validity as described by Penfold and Zhang^14^ (history, instrumentation, and selection bias) were rigorously assessed and mitigated, and all model assumptions were met.

### Meta-analysis

The RR estimates of disease and 95% CIs in the northern and southern hemispheres were combined with an inverse-variance weighted, fixed-effects meta-analysis to generate a pooled RR and 95% CI estimate. These models used a restricted maximum likelihood approach for coefficient estimates.^15^ This approach was also applied to pool the results of the various sensitivity analyses.

### Sensitivity analysis

For each of the four pathogens, segmented regression models were fitted to each country, stratified by age group and serotype or group. Negative binomial and quasi-Poisson generalised linear models were fitted to account for overdispersion of data, using population size as an offset and adjusting for seasonality, using month as a factor variable and Fourier terms.^13^ These models included a step change variable for implementation of containment measures, as described above. We also included models that extrapolated a counterfactual trend from pre-pandemic data. Further details are provided in the appendix (pp 1–2) and have been published previously.^4^

### Role of the funding source

The funders had no role in data collection, data analysis, data interpretation, writing of the manuscript, or the decision to submit the manuscript for publication.

## Results

All 30 countries participating in the IRIS Consortium submitted data on cases of *S pneumoniae* invasive disease, as represented by bacterial isolates or case reports, or both, submitted to each of the IRIS laboratories. Most countries also submitted invasive disease data on *H influenzae* (n=24) and *N meningitidis* (n=21), and nine countries submitted invasive disease data on *S agalactiae*. Overall, 116 841 cases were analysed: 76 481 in 2018–19, before the pandemic, and 40 360 in 2020–21, during the pandemic. The number of *S pneumoniae, H influenzae*, and *N meningitidis* cases during 2020–21 was approximately half the expected number each year compared to pre-pandemic totals, but the number of *S agalactiae* cases was similar each year (table 1).

**Table 1:** Overall number of invasive disease cases submitted to IRIS-participating laboratories before (2018–19) and during (2020–21) the COVID-19 pandemic

	**Streptococcus pneumoniae**	**Haemophilus influenzae**	**Neisseria meningitidis**	**Streptococcus agalactiae**
**2018**				
Number	30 553	3510	2302	1702
Median (IQR)	2405 (1824–3162)	282 (248–360)	192 (162–207)	147 (130–155)
**2019**				
Number	30 606	3697	2228	1883
Median (IQR)	2574 (2018–3050)	323 (273–342)	182 (164–196)	154 (144–171)
**2020**				
Number	15 501	2120	976	1886
Median (IQR)	834 (627–1258)	115 (102–169)	40 (36–81)	157 (145–168)
**2021**				
Number	15 306	2117	550	1904
Median (IQR)	1160 (1003–1391)	158 (128–194)	40 (35–56)	156 (149–170)

Data shown are numbers of isolates per year. The year corresponds to the International Organization for Standardization year. IRIS=Invasive Respiratory Infection Surveillance.

**Figure 1:**
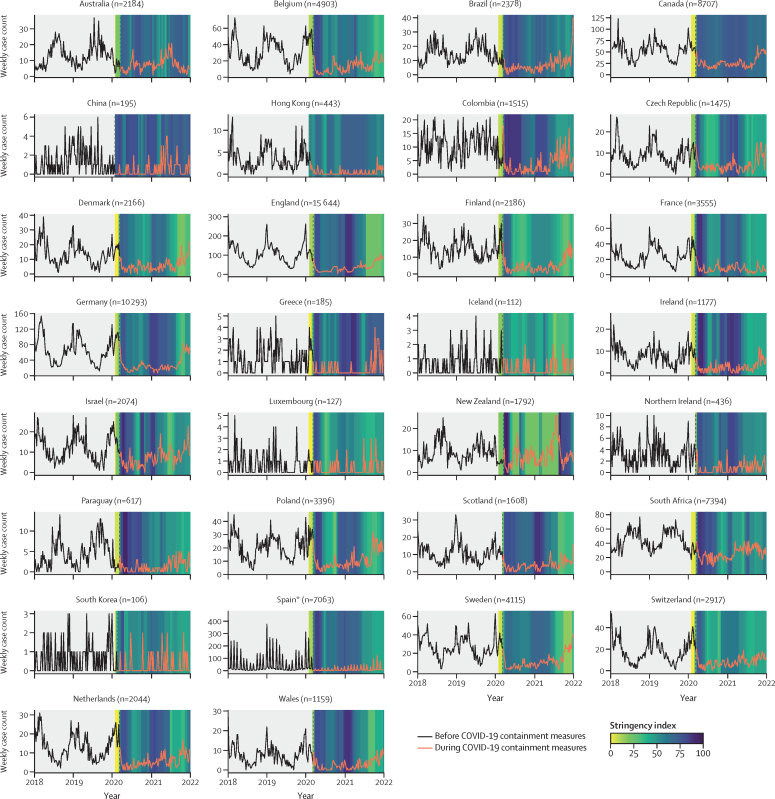
*Streptococcus pneumoniae* invasive disease case counts For each country or territory, weekly invasive disease cases from Jan 1, 2018, to Jan 2, 2022 (four complete International Organization for Standardization years), were plotted against the weekly Oxford COVID-19 Government Response Tracker stringency index value in 2020–21. The vertical dashed line indicates the week in which pandemic response measures were initiated in each country. *Many of the Spanish sampling dates were submitted only by month and not day of sampling, so the sampling date was entered as the first day of the month if the actual sampling day was unavailable.

There was an association between the stringency of COVID-19 containment measures implemented in each country and the number of *S pneumoniae* cases reported to laboratories (ie, as the stringency of containment measures decreased during 2021 in many countries, there were concomitant increases in *S pneumoniae* cases; figure 1). Similar associations were observed for *H influenzae* and *N meningitidis*, but not *S agalactiae* (appendix pp 3–5).

**Figure 2:**
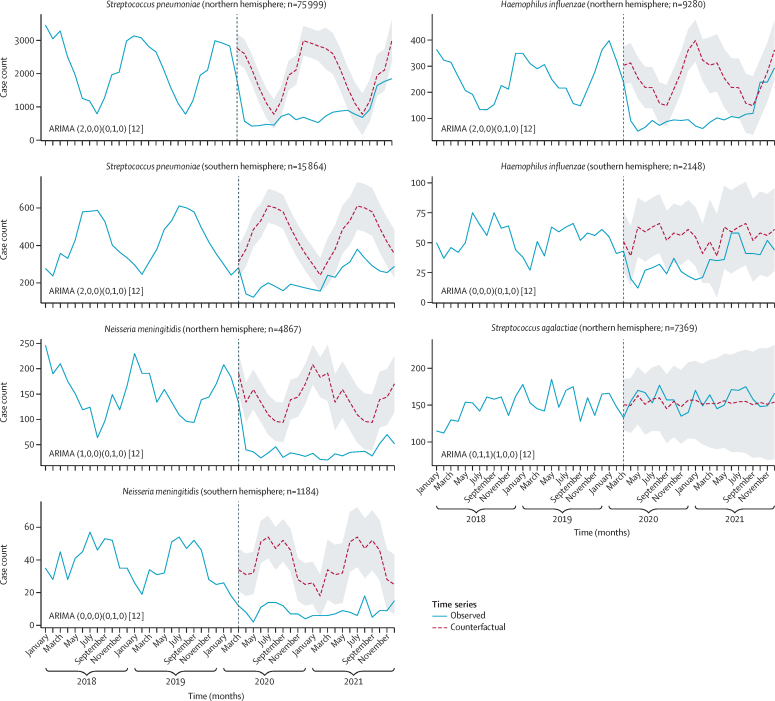
Interrupted time-series analyses of invasive disease data in the northern and southern hemispheres Observed cases of invasive disease for each bacterial species (blue solid lines) were plotted against the counterfactual weekly number of cases predicted by the ARIMA models (red dashed lines) if the COVID-19 pandemic had not occurred. The black vertical dashed line indicates the modal month (ie, the most common month when containment measures were put in place). The grey shading depicts 95% prediction intervals. *Streptococcus agalactiae* data were only collected in the northern hemisphere, and data are plotted by weeks in the calendar year rather than International Organization for Standardization year. The numbers next to ARIMA in the first set of parentheses indicate which components have been included to generate the counterfactual (p,d,q), while the second set of parentheses provides an indication of the seasonal model used (P,D,Q). The square brackets indicate that the model is generated using monthly data (12 months in a year). Please see text for details. ARIMA=autoregressive integrated moving average.

Time-series analyses by northern and southern hemispheres for each of the bacterial species showed an overall reduction in cases of disease caused by *S pneumoniae, H influenzae*, and *N meningitidis*, but not *S agalactiae,* during the pandemic (figure 2). Notably, cases due to *S pneumoniae, H influenzae*, and *N meningitidis* were increasing by the end of 2021. Google CCMR data were used to assess the point at which there was a step change that precipitated the reduction in cases of disease within each country, which was from March, 2020 (when the pandemic was declared), in both the northern and southern hemispheres (figure 2).

**Figure 3:**
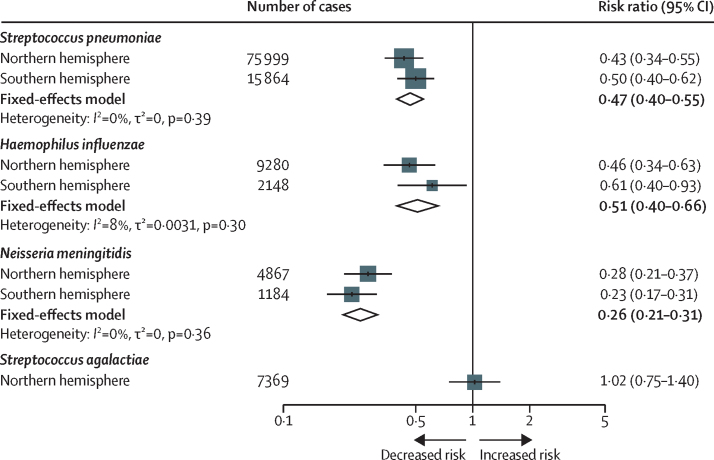
Risk of invasive disease during the pandemic for each bacterial species by hemisphere Results of the meta-analysis are shown as fixed-effects model estimates for each bacterial species.

The data were meta-analysed by hemisphere, the results of which showed a significant reduction in the risk of invasive disease caused by *S pneumoniae* (RR 0·47; 95% CI 0·40–0·55)*, H influenzae* (0·51; 0·40–0·66), and *N meningitidis* (0·26; 0·21–0·31) but not *S agalactiae* (1·02; 0·75–1·40; figure 3). Sensitivity analyses supported the use of the ARIMA model (appendix p 6), which estimated that 36 289 (95% PI 17 145–55 434) cases were averted in these 30 countries during the first 2 years of the pandemic (table 2).

**Table 2:** Estimated number of invasive disease cases averted during the COVID-19 pandemic (2020–21), by hemisphere

	**Cases averted (95% PI)**
**Streptococcus pneumoniae**	
Northern hemisphere	24 893 (13 377 to 36 410)
Southern hemisphere	5067 (2726 to 7409)
**Haemophilus influenzae**	
Northern hemisphere	3027 (966 to 5089)
Southern hemisphere	479 (−162 to 1120)
**Neisseria meningitidis**	
Northern hemisphere	2258 (1240 to 3275)
Southern hemisphere	649 (312 to 986)
**Streptococcus agalactiae**	
Northern hemisphere	−84 (−1314 to 1145)

Overall, 36 289 (95% PI 24 229 to 48 349) cases were averted. PI=prediction interval.

**Figure 4:**
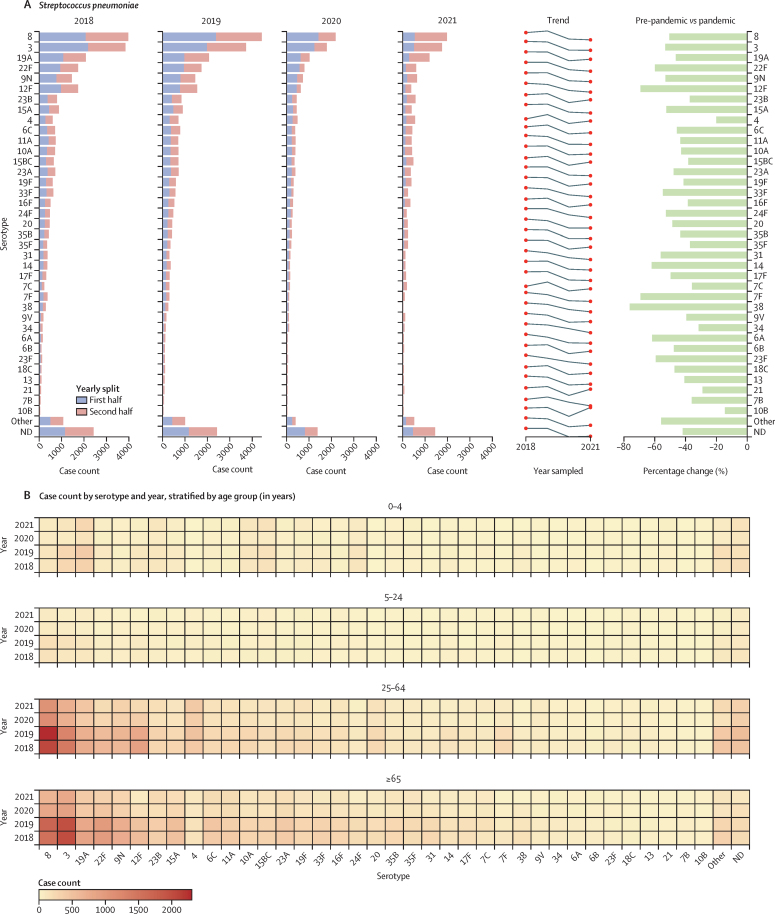
*Streptococcus pneumoniae* invasive disease cases by serotype and patient age (A) Distribution of serotypes responsible for 90% of all reported cases between 2018 and 2021, listed by case count, increasing or decreasing trend year by year, and percentage change of each serotype recovered in 2018–19 compared to 2020–21 (average number of cases each year, pre-pandemic *vs* during the pandemic). (B) Heat map depicting the number of cases of each serotype recovered per year and by age group. ND=not determined.

Data were then stratified by serotype or group and patient age. Among *S pneumoniae* cases, there were significant reductions in the case count of all major serotypes in 2020–21, although cases of disease due to some serotypes were beginning to increase in the latter months of 2021 (Figure 2, Figure 4). Case numbers in 2020–21 were reduced in every age category and there were no major changes in the overall patterns of disease by age or serotype (figure 4B).

**Figure 5:**
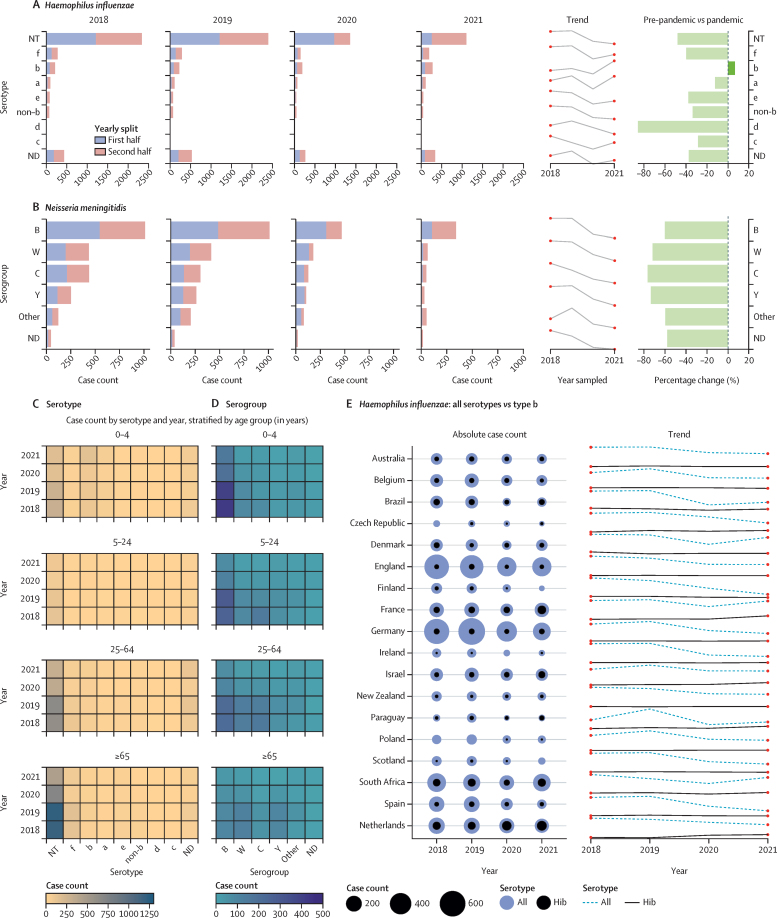
*Haemophilus influenzae and Neisseria meningitidis* invasive disease cases by serotype, capsule group, country, and age Distribution of serotypes between 2018 and 2021 for *Haemophilus influenzae* (A) and *Neisseria meningitidis* (B) are shown, listed by case count, increasing or decreasing trend year by year, with percentage change of each serotype recovered in 2018–19 compared to 2020–21 (average number of cases each year, pre-pandemic *vs* during the pandemic). Heat maps depicting the number of cases of each serotype recovered per year and by age group are shown for *H influenzae* (C) and *N meningitidis* (D). Number of cases of *H influenzae* by country are shown (E), displaying only those countries where at least 100 cases in total had been reported across all four study years. Circles represent the total number of cases each year, and lines indicate the increasing or decreasing trend year by year, with all serotypes depicted by the dashed line and *H influenzae* serotype b (Hib) depicted by the solid line. ND=not determined.

Stratification of *H influenzae* cases by serotype and patient age showed a reduction in case counts of all serotypes except for serotype b (Hib), which decreased in 2020 and then increased at the end of 2021 (p<0·0001). However, the total number of Hib cases remained very low: only 276 Hib cases were reported among 24 countries in 2021 (figure 5A). Overall, Hib cases increased among children aged 0–4 years in 2021 (n=146) versus 2020 (n=87; figure 5C). When stratified by country, the increase in Hib infections was primarily observed in five countries in 2021: the Netherlands (n=70), France (n=50), South Africa (n=43), Israel (n=27), and Paraguay (n=10; figure 5E).^6^, ^16^ Among cases of *N meningitidis* there was a significant reduction in infections due to all serogroups (but especially capsule groups W, C, and Y), with no obvious changes in the patterns of disease by age group (figure 5B, D).

## Discussion

Our statistical models estimated that, on average, more than 36 000 cases of life-threatening invasive bacterial diseases caused by *S pneumoniae, H influenzae*, and *N meningitidis* were averted in the countries participating in the IRIS Consortium during the first 2 years of the COVID-19 pandemic. The large number of averted cases reduced the morbidity and mortality associated with these infections, which would have eased the burden on some health-care systems during the pandemic. The documented reduction in infections was most plausibly due to the worldwide implementation of COVID-19 containment measures aimed at reducing transmission of SARS-CoV-2, which simultaneously reduced transmission of other microbes that spread via respiratory secretions, including those studied by the IRIS Consortium.

Our findings cannot be explained by under-reporting of data by hospitals and laboratories that were overwhelmed by the pandemic. In most of the 30 countries and territories represented in this analysis, it is a legal requirement to report cases of invasive disease caused by the pathogens covered in this study, and the inclusion of *S agalactiae* as a non-respiratory comparator organism provided reassurance that the surveillance programmes in these laboratories were functioning without major disruptions during the pandemic.

The findings show that although cases of disease remained significantly lower in 2020–21 compared to pre-pandemic levels, rates of disease were increasing in some countries and territories towards the end of 2021.^17^, ^18^ It is therefore reasonable to predict that rates of invasive bacterial infections will return to pre-pandemic levels in due course. Furthermore, the 2021 data showed a small overall increase in Hib cases after an initial decrease in 2020. However, other factors could have influenced these dynamics even before the pandemic, such as changes to Hib vaccines or vaccine schedules (or both), or pre-pandemic increases in the incidence of Hib infection.^6^, ^16^ Renewed emphasis on the active surveillance of invasive diseases caused by Hib is certainly warranted going forward.

An important concern now, as people have returned to normalised social interactions, is which pathogens will cause disease. The usual patterns of microbial transmission were altered during the pandemic, and if the microbiome within the upper respiratory tract was also disrupted, this could lead to changes in the prevalence of serotypes or groups associated with disease, increased prevalence of circulating non-vaccine types, or emergence of non-traditional disease-associated types.^19^, ^20^ If the microbiome was not substantially disrupted, then one might expect a return to disease patterns that are recognisable to those observed before the pandemic. Time will reveal which of these outcomes proves to be true.

There is also growing concern around decreased population immunity or a so-called immunity debt (ie, a higher proportion of susceptible individuals within a population because of reduced exposure to commonly circulating microbes) as a result of pandemic restrictions, which could lead to future outbreaks of disease.^21^, ^22^ Certain populations might be at increased risk of infection, such as children born during the pandemic; teenagers and young adults because of their increased social mixing; and older people because of immunosenescence, high rates of underlying comorbidities, and frailty. The situation is further compounded by disruptions in routine vaccination schedules around the world since health-care systems were reorganised to deal with the threat of the pandemic, but at the expense of providing other essential public health services.^23^, ^24^, ^25^, ^26^ For example, reinstatement of routine paediatric vaccination programmes is one of the most important post-pandemic challenges that remains to be addressed in many parts of the world.

In addition to reduced exposure to pathogens, the typical patterns of respiratory disease were disrupted during the pandemic, and the prevalence of commonly circulating respiratory viruses such as respiratory syncytial virus and influenza viruses was also reduced during the pandemic due to the implementation of COVID-19 containment measures.^27^, ^28^ Two recent studies reported a correlation between the reduced prevalence of respiratory viruses and reductions in diseases due to *S pneumoniae* during the pandemic.^29^, ^30^ Further work will be necessary to better understand any causative relationship between respiratory viruses and colonising nasopharyngeal bacteria, mechanisms of co-infections, and to mechanistically understand how one microbe might influence the pathogenicity of another.

Limitations of the time-series analyses in this study included difficulties in fitting ARIMA models to individual countries and territories. Each country has its own pattern of disease and public health restrictions, and some countries reported relatively small case numbers. This necessitated temporal pooling by month and geographical pooling by hemisphere, leading to wider prediction intervals and reduced predictive power of the models. Although we assessed data from 2018 to 2021, when taking account of a yearly trend, the necessary seasonal adjustment in the model leads to a loss of a year's worth of data, which affected our sample size.^12^

Despite these limitations, strengths of these analyses included the large datasets rapidly contributed by investigators, which spanned 4 years and increased the power of these analyses. Additionally, high-quality data at a national level were made available by accredited reference laboratories undergoing routine audit and data validation practices, which minimised information bias. Selection bias is likely to be minimal because the bacteria under investigation cause diseases that necessitate urgent hospital care, and legislation in most of these countries and territories mandates the reporting of invasive diseases due to one or more of these bacteria. We also tested a range of time-series analyses to ensure the robustness of the results and the findings were reproducible.

As our societies emerge from the COVID-19 pandemic, this large prospective study by the IRIS Consortium allows for timely detection of changes in invasive diseases caused by *S pneumoniae, H influenzae*, and *N meningitidis*, and provides a means to detect and address substantial changes that will undoubtedly occur. Most importantly, it is essential that any ongoing disruptions to bacterial vaccination programmes are resolved since diseases due to these bacteria are devastating but can be prevented by safe and effective vaccines already used in many countries worldwide.

## Data sharing

Sharing of study data is not possible because of the risk of identifying individual cases of invasive disease in some countries. The source code for the statistical analyses is available via GitHub.

## Declaration of interests

The UK Health Security Agency's Immunisation and Vaccine Preventable Diseases Division has provided vaccine manufacturers (GSK, Pfizer, and Sanofi) with post-marketing surveillance reports, which the Marketing Authorization Holders are required to submit to the UK Licensing authority in compliance with their Risk Management Strategy. A cost recovery charge is made for these reports. The UK Health Security Agency's Respiratory and Vaccine Preventable Bacteria Reference Unit has received unrestricted research grants from Pfizer to participate in pneumococcal surveillance projects. CHI de Créteil (France) received research grants from the French Public Health Agency, Pfizer, and MSD. University Hospitals Leuven (Belgium) received research grants from Merck-MSD and Pfizer, and consulting fees from Merck-MSD. SD received personal payments or honoraria from Pfizer. The Swiss National Reference Center for Invasive Pneumococci received funding from the Federal Office of Public Health. MH has received grants from Pfizer and personal fees (for being on an advisory board) from Pfizer and Merck Sharp & Dohme. The National Medicines Institute (Warsaw, Poland) received funding from the Polish Ministry of Health, the Polish Ministry of Science and Higher Education, Pfizer, and MSD. AS received payments from MSD and Pfizer for lectures, and from MSD, Pfizer, and Sanofi Pasteur for participation in advisory boards. AS is the unpaid Vice President of the European Meningococcal and Haemophilus Disease Society. The Finnish Institute for Health and Welfare (Finland) received research funding from Pfizer. ABB is an unpaid adviser to WHO, providing expertise related to vaccines and antimicrobial resistance. ABB is an unpaid General Assembly member (2022 onwards), and has been a board member (2016–22) and Secretary (2018–22), of the International Society of Pneumonia and Pneumococcal Diseases (ISPPD). MD has received financial support from Pfizer to attend national scientific meetings. MdP received grant funding from the National Research Foundation (South Africa) and the Bill & Melinda Gates Foundation to support the International Pathogenic Neisseria Conference (IPNC) 2022 meeting. MdP received personal support from the ISPPD to participate in the ISPPD conference in 2022, and was a member of the organising and scientific committee for the IPNC meeting in 2022. HH and MC received a grant from Pfizer (W1243730) to investigate Irish pneumococcal serotypes by whole-genome sequencing. HH received payment from Scottish Hospitals Enquiry for expert testimony. HH was the President of the Healthcare Infection Society (2018–22). KAJ received personal royalties from GlaxoSmithKline, and personal honoraria from the Wellcome Trust. SL performs contract research on behalf of St George's University of London for pharmaceutical companies (GlaxoSmithKline, Pfizer, and Sanofi), including vaccine manufacturers, but does not receive any personal remuneration. T-TL received consulting fees from the Trond Mohn Foundation. T-TL is an unpaid board member of the European Meningococcal and Haemophilus Disease Society and the German Society for Hygiene and Microbiology, committee for microbial systematics, population genetics and infection epidemiology. SM participated on an unpaid advisory board for Pfizer for the meningococcal type B vaccine in South Africa in 2020. WM received funding from GlaxoSmithKline and Pfizer for investigator-initiated research on meningitis B (MenB) strain vaccine coverage. CS received financial support for flights, accommodation, and registration to attend the 2022 ISPPD meeting in Canada. H-CS received funding from Pfizer for a pneumococcal carriage project. H-CS received funding for participation on a data safety monitoring board or advisory board for MSD. LS received personal support from the European Centre for Disease Prevention and Control for attending the European Scientific Conference on Applied Infectious Disease Epidemiology in 2022. MPGvdL received consulting fees from Pfizer, Merck, and GlaxoSmithKline; payment or honoraria from Pfizer and Merck; and support for attending meetings or travel, or both, from Pfizer. AvG is the chairperson for the National Advisory Group on Immunization of South Africa. NMvS received fees for services and consulting fees from MSD and GlaxoSmithKline, and research funding from the Dutch Health Counsel, US National Institutes of Health, and Amsterdam University Medical Centers, and from MSD and GlaxoSmithKline, which are all directly paid to the institution. NMvS holds a patent (WO 2013/020090 A3) on vaccine development against *Streptococcus pyogenes.* NMvS is an unpaid scientific adviser to the ItsME foundation, and a scientific adviser to the StrepAotearoa New Zealand project but fees are paid to the University of Amsterdam. NMvS holds personal stocks in Genmab. JY received payments for lectures given at scientific meetings organised by MSD and Pfizer; received support from MSD and Pfizer to attend national and international scientific meetings; and participated in advisory boards for MSD and Pfizer. DS is supported by an Oxford Clarendon Scholarship. All other authors declare no competing interests.
