# Supplementary material for: Trends in invasive bacterial diseases during the first 2 years of the COVID-19 pandemic: analyses of prospective surveillance data from 30 countries and territories in the IRIS Consortium
Source: Lancet Digit Health. 2023 Jul 27;5(9):e582–93. doi: 10.1016/S2589-7500(23)00108-5 (PMC10914672; doi:10.1016/S2589-7500(23)00108-5)

THE LANCET
Digital Health

Supplementary appendix

This appendix formed part of the original submission and has been peer reviewed. We post it as supplied by the authors.

Supplement to: Shaw D, Abad R, Amin-Chowdhury Z, et al. Trends in invasive bacterial diseases during the first 2 years of the COVID-19 pandemic: analyses of prospective surveillance data from 30 countries and territories in the IRIS Consortium. *Lancet Digit Health* 2023; published online July 27. https://doi.org/10.1016/S2589-7500(23)00108-5.

Appendix

Shaw et al, Trends in invasive bacterial diseases during the first 2 years of the COVID-19 pandemic: analyses of prospective surveillance data from 30 countries and territories in the IRIS Consortium

Supplementary methods: ARIMA and segmented regression models, sensitivity analyses

As discussed by Schaffer et al, when performing an interrupted time series analysis, there are two broad modelling approaches: choosing ARIMA models or segmented regression models. They argue that ARIMA models are a statistically more robust approach since ARIMA models take account of autocorrelation in time series data, while segmented regression approaches do not and this leads to inaccurate model estimates. Despite this, segmented regression approaches are widely used to conduct interrupted time series analyses. We decided to use ARIMA models as our main approach and built segmented regression models as a sensitivity analysis to assess whether the two approaches gave similar results.

We followed the segmented regression approach described by Bernal et al. In brief, our segmented regression models are generalised linear models either from the quasi-Poisson or negative binomial family. These models include a dummy variable to indicate when pandemic containment measures were put in place (ie intervention). The segmented regression models take the mathematical form shown below.

We included Fourier terms or treated the month of the year as a categorical (factor) variable to model the seasonality in the time series and we varied our assumptions of how the data were distributed by running models from different families to take account of overdispersion in the data. These models were built for each country and each organism; pooled results were obtained using inverse variance-weighted meta-analysis.

The timing of containment measure intervention was ascertained using a combination of Google Community mobility data and the Oxford Blavatnik COVID-19 government response tracker stringency index. Additionally, population size data for each year (2018-2021) from the World Bank and the UK Office of National Statistics (ONS) were used as model offsets. The intervention dates were detailed in our previous paper (Brueggemann et al).

References:

- Schaffer AL, Dobbins TA, Pearson SA. Interrupted time series analysis using autoregressive integrated moving average (ARIMA) models: a guide for evaluating large-scale health interventions. BMC Med Research Methodol 2021; 21(1):1-2.

- Bernal JL, Cummins S, Gasparrini A. Interrupted time series regression for the evaluation of public health interventions: a tutorial. Intl J Epidemiol 2017; 46(1):348-55.

- Brueggemann AB, Jansen van Rensburg MJ, Shaw D, et al. Changes in the incidence of invasive disease due to *Streptococcus pneumoniae*, *Haemophilus influenzae*, and *Neisseria meningitidis* during the COVID-19 pandemic in 26 countries and territories in the Invasive Respiratory Infection Surveillance Initiative: a prospective analysis of surveillance data. Lancet Digit Health. 2021; 3(6):e360-e370. doi: 10.1016/S2589-7500(21)00077-7.

A.1 Segmented Regression Models

A.1.1 With Fourier Terms

$$\ln(count_t) = \beta_0 + \beta_1 T_t + \beta_2 sine(\frac{2\pi m_t}{12}) + \beta_3 cosine(\frac{2\pi m_t}{12}) + \beta_4 sine(\frac{\pi m_t}{12}) + \beta_5 cosine(\frac{\pi m_t}{12}) + \beta_6 I_t + \epsilon_t \quad (A.1)$$

A.1.2 Without Fourier Terms

$$\ln(count_t) = \beta_0 + \beta_1 T_t + \beta_2 M_t + \beta_3 I_t + \epsilon_t \quad (A.2)$$

where:

$count_t$ is the number of cases in month t

T is the time in months starting from January 2018

m is the month of the year

M is the month in a 12-month period (seasonal adjustment)

I_t is the intervention step function:

$$I_t = 0 \text{ when } t < T_i$$
$$I_t = 1 \text{ when } t \geq T_i$$

T_i represents the time of intervention implementation

ϵ_t is the error term following either a quasi-Poisson or a negative binomial distribution

Supplementary Figure 1. *H influenzae* invasive disease case counts. For each country, weekly invasive disease cases from 1 January 2018 to 2 January 2022 (four complete ISO years) were plotted against the weekly Oxford COVID-19 Government Response Tracker stringency index value in 2020-2021. The vertical hashed line indicates the week in which pandemic response measures were initiated in each country.

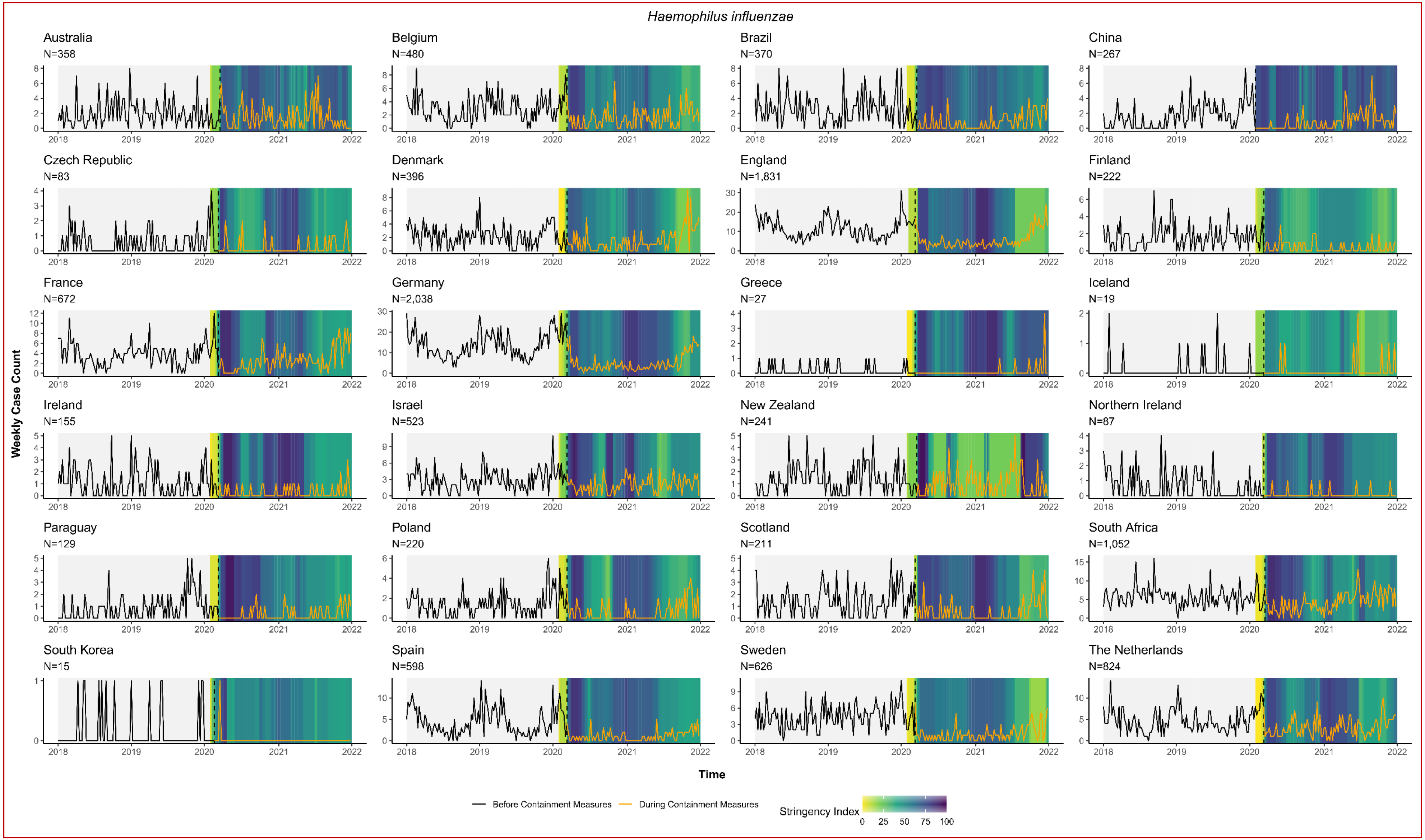

Supplementary Figure 2. *N meningitidis* invasive disease case counts. For each country, weekly invasive disease cases from 1 January 2018 to 2 January 2022 (four complete ISO years) were plotted against the weekly Oxford COVID-19 Government Response Tracker stringency index value in 2020-2021. The vertical hashed line indicates the week in which pandemic response measures were initiated in each country.

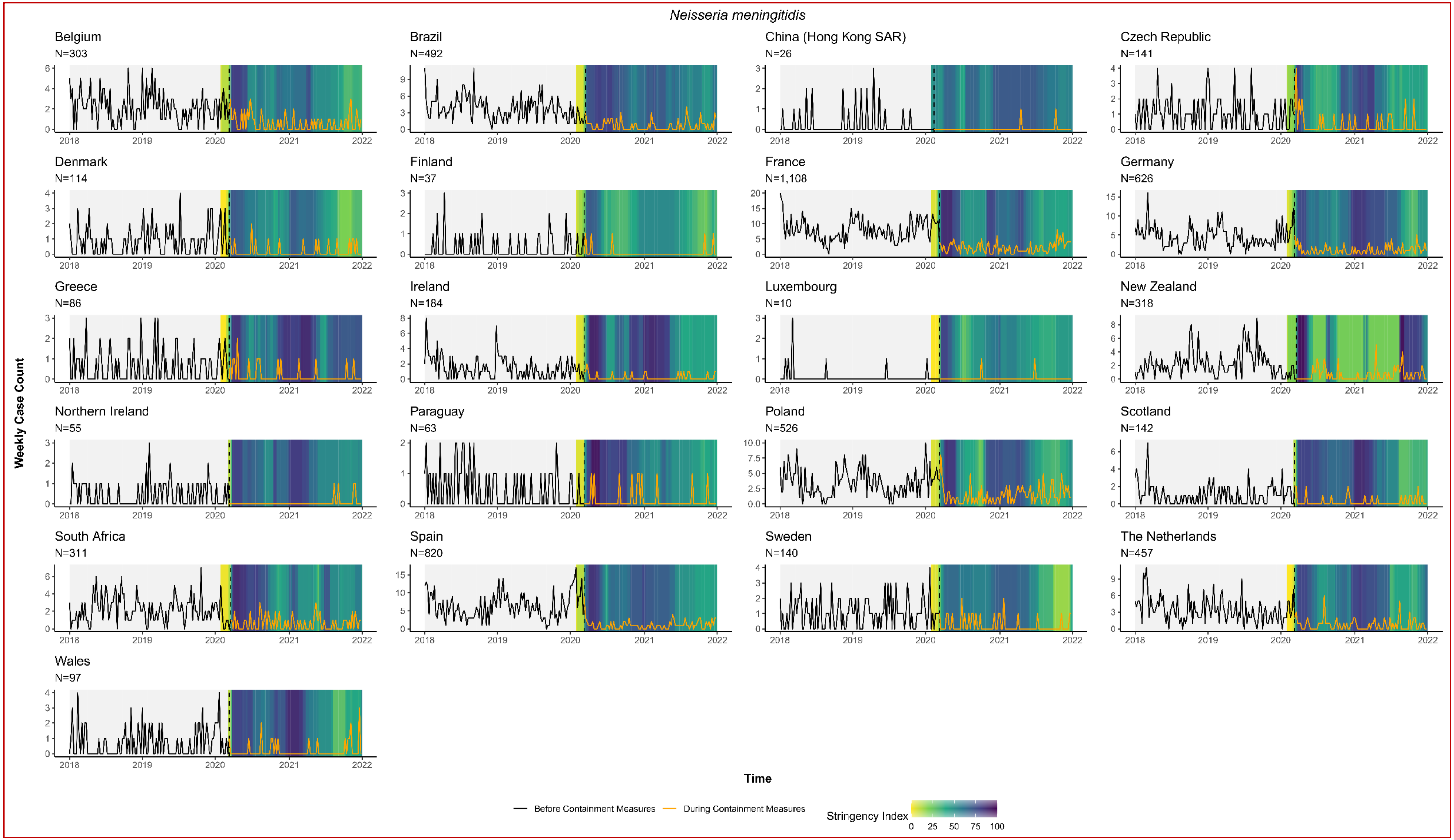

Supplementary Figure 3. *S agalactiae* invasive disease case counts. For each country, weekly invasive disease cases from 1 January 2018 to 2 January 2022 (four complete ISO years) were plotted against the weekly Oxford COVID-19 Government Response Tracker stringency index value in 2020-2021. The vertical hashed line indicates the week in which pandemic response measures were initiated in each country.

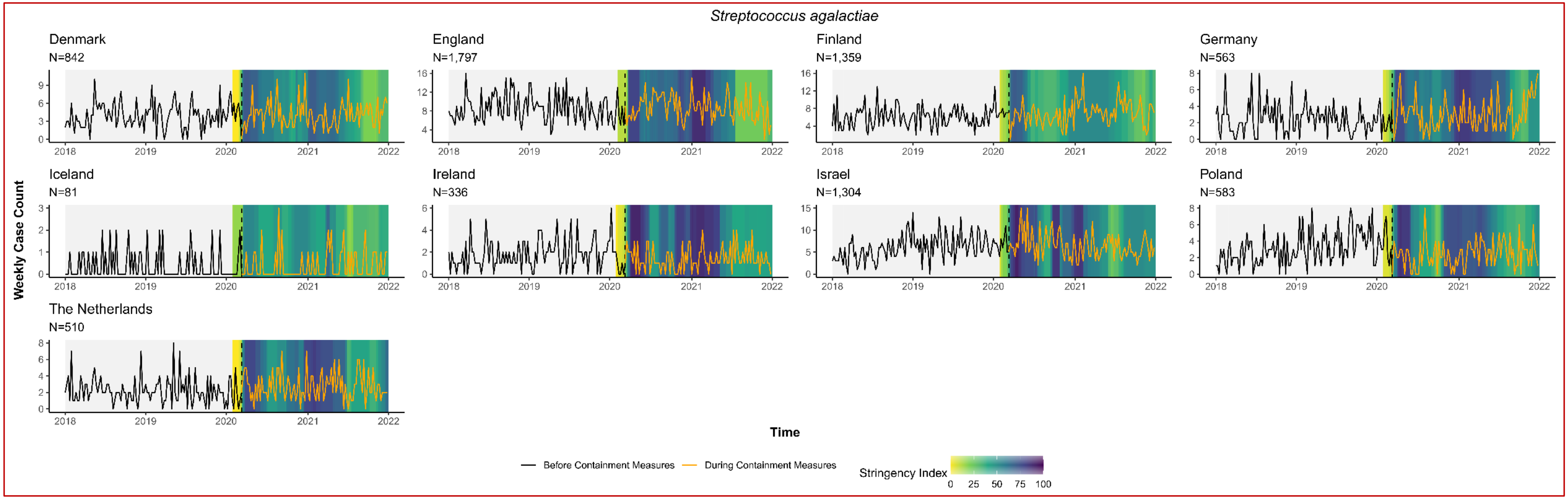

Supplementary figure 4. Sensitivity analyses comparing the effect of applying different models to the IRIS data.

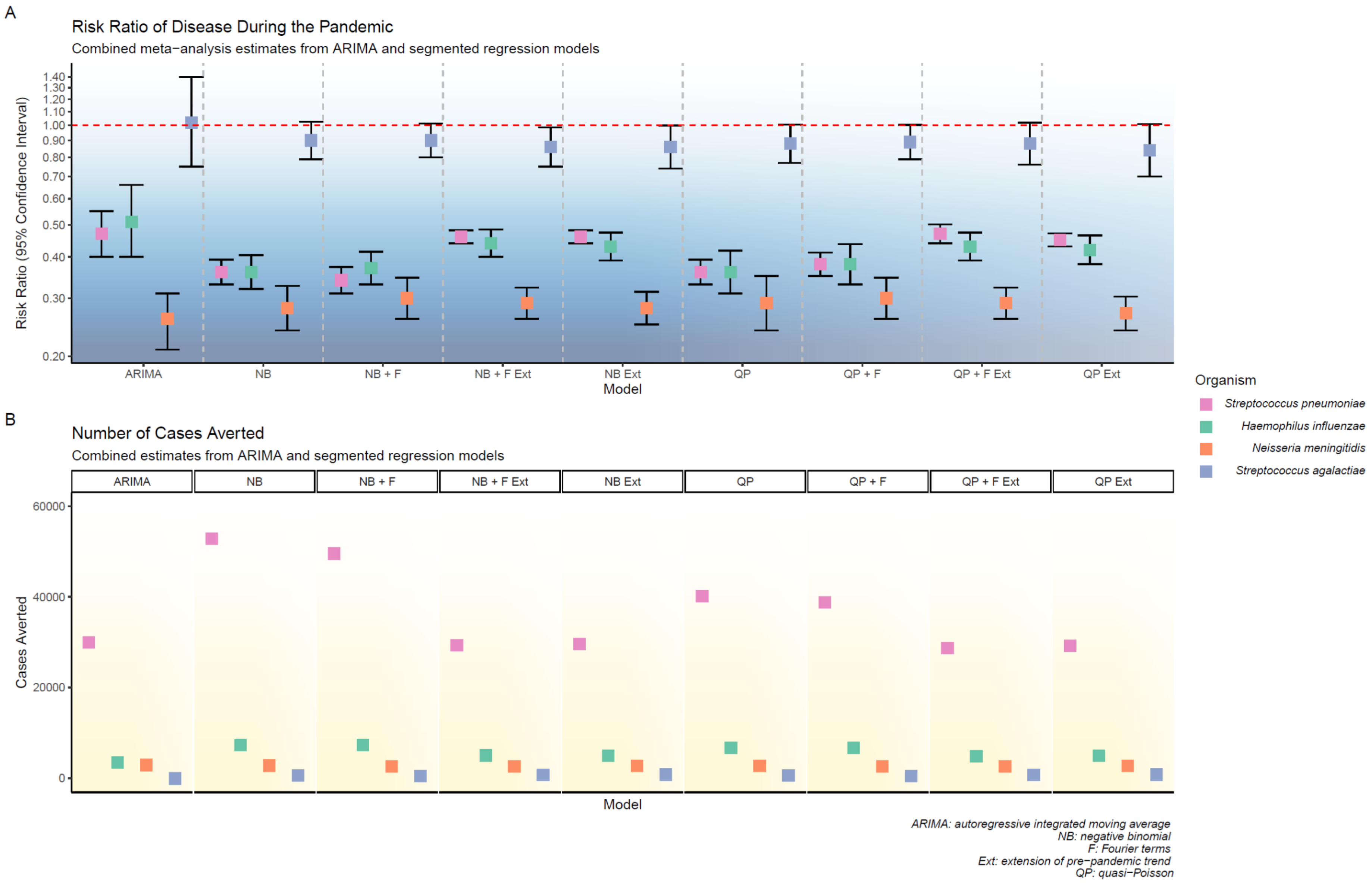

Supplement: Supplementary appendix [file mmc1.pdf]
